# Supplementary material for: Structural basis for diguanylate cyclase activation by its binding partner in Pseudomonas aeruginosa
Source: eLife. 2021 Sep 9;10:e67289. doi: 10.7554/eLife.67289 (PMC8457831; doi:10.7554/eLife.67289)
Supplement: Supplementary file 1. — (a) Strains and plasmids used in this study. (b) Primers used in this study. (c) Data collection and refinement statistics. (d) Data collection and refinement statistics. (e) Estimated secondary structure content of SiaD. [file elife-67289-supp1.docx]

**Supplementary materials for:**

**Structural basis for diguanylate cyclase activation by its binding partner in *Pseudomonas aeruginosa***

Gukui Chen^1, #^, Jiashen Zhou^2, #^, Yili Zuo^1^, Weiping Huo^1^, Juan Peng^1^, Meng Li^1^, Yani Zhang^1^, Tietao Wang^1^, Lin Zhang^2^, Liang Zhang^2,*^ and Haihua Liang^1,*^

^1^Key Laboratory of Resources Biology and Biotechnology in Western China, Ministry of Education, College of Life Sciences, Northwest University, Xi'an, ShaanXi, 710069, China

^2^ Department of Pharmacology and Chemical Biology, Shanghai JiaoTong University, School of Medicine, Shanghai, China, 200025

^#^These authors contributed equally to this work

*To whom correspondence should be addressed:

Dr. Haihua Liang

College of Life Science, Northwest University, No 229 Taibai Rd, Xi’an, ShaanXi 710069, China; Phone: (86) 029-88303662. Email: [lianghh@nwu.edu.cn](mailto:lianghh@nwu.edu.cn)

Dr. Liang Zhang

Shanghai Jiao Tong University School of Medicine, No 280 South Chongqing Road, Shanghai, China, 200025; Phone: (86) 021-54561256. Email: [liangzhang2014@sjtu.edu.cn](mailto:liangzhang2014@sjtu.edu.cn)

**Supplementary file 1a. Strains and plasmids used in this study.**

| Strains or plasmids | **Relevant characteristics or function** | **Reference or origin** |
| --- | --- | --- |
| **Plasmids** |  |  |
| pUCP20 | *Escherichia-Pseudomonas* shuttle vector | *(West, Schweizer et al., 1994)* |
| pUCP20-*siaC* | pUCP20 derived plasmid expressing *siaC*; Amp^r^ | This lab |
| pUCP20-*siaC^N67A/T68A/S69A^* | pUCP20 derived plasmid expressing *siaC^N67A/T68A/S69A^*; Amp^r^ | This study |
| pUCP20-*siaD* | pUCP20 derived plasmid expressing *siaD*; Amp^r^ | This lab |
| pUCP20-*siaD^D138A^* | pUCP20 derived plasmid expressing *siaD^D138A^*, Amp^r^ | This study |
| pUCP20- *siaD^D155A^* | pUCP20 derived plasmid expressing *siaD^D155A^*, Amp^r^ | This study |
| pUCP20- *siaD^E181A^* | pUCP20 derived plasmid expressing *siaD^E181A^*, Amp^r^ | This study |
| pUCP20- *siaD^K250A/R254A^* | pUCP20 derived plasmid expressing *siaD^K250A/R254A^*, Amp^r^ | This study |
| pUCP20-*siaD-flag* | pUCP20 derived plasmid expressing *siaD-flag*, Amp^r^ | This lab |
| pUCP20-*siaD^D138A^-flag* | pUCP20 derived plasmid expressing *siaD^D138A^-flag*, Amp^r^ | This study |
| pUCP20- *siaD^D155A^-flag* | pUCP20 derived plasmid expressing *siaD^D155A^-flag*, Amp^r^ | This study |
| pUCP20- *siaD^E181A^-flag* | pUCP20 derived plasmid expressing *siaD^E181A^-flag*, Amp^r^ | This study |
| pUCP20- *siaD^K250A/R254A-^flag* | pUCP20 derived plasmid expressing *siaD^K250A/R254A-^flag*, Amp^r^ | This study |
| pMMB67EH | *Escherichi-Pseudomonas* Shuttle vector between; Amp^r^ | S. Jin lab |
| pMMB67EH-*flag* | pMMB67EH vector with Flag tag coding sequence | *(Chen, Gan et al., 2020)* |
| pMMB67EH-*siaC*-*flag* | pMMB67EH-Flag derived plasmid expressing *siaC-flag* | This study |
| pMMB67EH-*siaC^N67A/T68AS69A^*-*flag* | pMMB67EH-Flag derived plasmid expressing *siaC^N67A/T68AS69A^*-Flag | This study |
| pMMB67EH-*siaD*-*flag* | pMMB67EH-Flag derived plasmid expressing *siaD-flag* | This study |
| pMMB67EH-*siaD^ΔN40^*-*flag* | pMMB67EH-Flag derived plasmid expressing *siaD^ΔN40^-flag* | This study |
| pMMB67EH-*siaD^ΔN95^*-*flag* | pMMB67EH-Flag derived plasmid expressing *siaD^ΔN95^-flag* | This study |
| pGEX-*siaD* | Plasmid for GST-SiaD expression | This study |
| pET-Sumo | Plasmid for Sumo expression | *(Yang, Wu et al., 2018)* |
| pSumo-*siaD* | Plasmid for Sumo-SiaD expression | This study |
| pSumo-*siaC* | Plasmid for Sumo-SiaC expression | This lab |
| pSumo-*siaD^ΔN40^* | Plasmid for Sumo-SiaD^ΔN40^ expression | This study |
| pSumo-*siaD^D138A^* | Plasmid for Sumo-SiaD^D138A^ expression | This study |
| pSumo-*siaD^E181A^* | Plasmid for Sumo-SiaD^E181A^ expression | This study |
| pSumo-*siaC^N67A/T68A/S69A^* | Plasmid for Sumo-SiaC^N67A/T68A/S69A^ expression | This study |
| **Strains** |  |  |
| ***P. aeruginosa*** |  |  |
| PAO1 | Wild type | This lab |
| ∆*siaC* | *siaC* deletion mutant of PAO1 | This lab |
| ∆*siaD* | *siaD* deletion mutant of PAO1 | This lab |
| ***E. coli*** |  |  |
| DH5α | F– *φ80lacZ* Δ*M15* Δ(*lacZYA-argF)U169 recA1 endA1 hsdR17(rk–, mk+)phoA supE44 thi-1 gyrA96 relA1 tonA* | Invitrogen |
| BL21(DE3) | F- *ompT hsdS_B_(r_B_^-^m_B_^-^) gal dcm* (DE3) | Invitrogen |

**Supplementary file 1b. Primes used in this study.**

| **Primer** | **Sequence (5'→3')^a^** | **Application** |
| --- | --- | --- |
| pUC-*siaC*-F | AGCgaattcCGTGTTTTAGGAAGAACACCATC | For constructing pUCP-*siaC* |
| pUC-*siaC*-R | CCCaagcttCTACTCGTCGTGGGCCTG | For constructing pUCP-*siaC* |
| *siaC*-m3-F | CTGTACCTGGCTGCCGCTTCGATCAAGGCCATG | For constructing pUC*-siaC^N67A/T68A/S69A^*, pSumo-*siaC^N67A/T68A/S69A^* and pMM- *siaC^N67A/T68A/S69A^* |
| *siaC*-m3-R | CATGGCCTTGATCGAAGCGGCAGCCAGGTACAG |  |
| pUC*-siaD*-F | AGCgaattcGGACCTGCGCCTGCTGTACC | For constructing pUCP-*siaD* |
| pUC-*siaD*-R | CCCaagcttTCAGCGCGCTGGAGCCGGG | For constructing pUCP-*siaD* |
| pUC-*siaD-flag*-R | CCCaagcttTCACTTGTCATCGTCGTCCTTGTAGTCGCGCGCTGGAGCCGGG | For constructing pUCP-*siaD-flag* |
| SiaD^D138A^-F | TGCTGGCAATGCTCGCTGTGGACTTCTTCAA | For constructing pUCP-*siaD^D138A^*-flag and pSumo-*siaD^D138A^* |
| SiaD^D138A^-R | TTGAAGAAGTCCACAGCGAGCATTGCCAGCA |  |
| SiaD^D155A^-F | GCCACGACAGCGGTGCCCGGGTGCTGGTGGA | For constructing pUCP-*siaD^D155A^*-flag and pSumo-*siaD^D155A^* |
| SiaD^D155A^-R | TCCACCAGCACCCGGGCACCGCTGTCGTGGC |  |
| SiaD^E181A^-F | GCCGCTGGGGCGGCGCGGAATTCCTCCTGCT | For constructing pUCP-*siaD^E181A^*-flag and pSumo-*siaD^D181A^* |
| SiaD^E181A^-R | AGCAGGAGGAATTCCGCGCCGCCCCAGCGGC |  |
| SiaD^K250A/R254A^-F | GCACTGCTCGACGCCGCGCGCAGCGGCGCCGACAAATGCGTGTT | For constructing pUCP-*siaD^K250A/R254A^*-flag and pSumo- *siaD^K250A/R254A^* |
| SiaD^K250A/R254A^-R | AACACGCATTTGTCGGCGCCGCTGCGCGCGGCGTCGAGCAGTGC |  |
| pmm-*siaC*-F | AGCgaattcATGAGTGACCTGCACATACCCG | For constructing pMMB67EH-*siaC*-Flag |
| pmm-*siaC*-R | CCCaagcttCTCGTCGTGGGCCTGGAT | For constructing pMMB67EH-*siaC*-Flag |
| pmm-siaD-F | AGCgagctcATGCGGCTGGAGCGCATCG | For constructing pMMB67EH-*siaD*-Flag |
| pmm-*siaD^ΔN40^*-F | AGCgagctcGTGCGGCTGGAGCGCATCG | For constructing pMMB67EH- *siaD^ΔN40^*-Flag |
| pmm-siaD95-F | AGCgagctcATGCTGAAGGAAGCCTCGATCC | For constructing pMMB67EH- *siaD^ΔN95^*-Flag |
| pmm-siaD-R | CCCaagcttGCGCGCTGGAGCCGGGCG | For constructing pMMB67EH-*siaD*-Flag |
| siaC-bam-F | TTAggatccGGCGGAGGAATGAGTGACCTGCAC | For constructing pSumo-*siaC* |
| siaC-eco-R | CCCgaattcCTACTCGTCGTGGGCC | For constructing pSumo-*siaC* |
| siaD-bam-F | ATAggatccGTGCGGCTGGAGCGCAT | For constructing pSumo-siaD |
| siaD-sal-R | CCCgtcgacGCGCGCTGGAGCC | For constructing pSumo-siaD |

**Supplementary file 1c. Data collection and refinement statistics.**

|  | SiaC-SiaD complex |
| --- | --- |
| **Data collection** |  |
| Space group | *C222_1_* |
| Cell dimensions |  |
| *a, b, c*(Å) | *82.525, 236.728, 148.627* |
| α, β, γ (°) | *90.00, 90.00, 90.00* |
| Wavelength (Å) | *0.9785* |
| Resolution (Å)* | *50.0-2.65(2.74-2.65)* |
| *R*_merge_ (%) | *22.2(171)* |
| *I*/σ*I* | *13.1(1.5)* |
| Completeness (%) | *99.5(99.5)* |
| Redundancy | *13.3(12.8)* |
| CC_1/2_ (%) | *98.2(58.8)* |
|  |  |
| **Refinement** |  |
| Resolution (Å) | *50-2.65* |
| No.reflections (percentage in Rfree test set) | *2535157 (5%)* |
| *R*_work_/*R*_free_ | *0.218/0.265* |
| No.atoms |  |
| Protein | *8251* |
| Water | *197* |
| Ligand/ion | *33* |
| B-factors |  |
| Protein | *55.6* |
| Water | *42.1* |
| Ligand/ion | *52.2* |
| R.m.s deviations |  |
| Bond lengths (Å) | *0.027* |
| Bond angles (°) | *1.749* |

*Highest-resolution shell is shown in parentheses.

**Supplementary file 1d.** SAXS parameters of SiaD and SiaC-SiaD Complex

| **Data Collection Parameters** | | |
| --- | --- | --- |
| Beam line | SSRF (Shanghai, China) BL19U2 | |
| Wavelength (Å) | 0.923 | |
| Detector | Pilatus 1M | |
| *q* range (Å ^-1^) | 0.009-0.45 | |
| Exposure time(s) | 1.5 for SEC-SAXS | |
| Concentration range (mg/ml) | 11.0 for SiaC-SiaD complex , 8.0 for SiaD | |
| Temperature (°C) | 23 | |
| **Structural Parameters** | **SiaC-SiaD complex** | **SiaD** |
| *I*(0) (cm^-1^) from Guinier fit | 134.26±0.36 | 50.90±0.20 |
| *R*g(Å) from Guinier fit | 44.54±0.17 | 42.81±0.34 |
| *I*(0) (cm^-1^) from *P*(*r*) | 134.90±0.25 | 50.27±0.17 |
| *R*g(Å) from *P*(*r*) | 45.68±0.08 | 41.53±0.14 |
| *D*max (Å) from *P*(*r*) | 134.8 | 127.8 |
| **Molecular Mass Determination** | | |
| MW (kDa) from SEC-SAXS | 105.7 | 159.5 |
| MW (kDa) from sequence | 121.6 | 157.3 |
| Using the volume of correlation(Vc) | 99.8 | 168.4 |
| MW from the *V*_porod_ | 109.5 | 165.2 |
| **Modeling** | |  |
| DAMMIN χ^2^ | 1.075 | 1.116 |
| DAMMIN Ensemble Resolution | 47±4 | 65±5 |
| DAMMIN NSD | 1.355±0.208 | 1.486±0.186 |
| **Software Employed** | | |
| Primary data Processing | RAW | |
| P(r) | GNOM | |
| *Ab initio* shape analysis | DAMMIF | |
| SAXS Profile computation | CRYSOL | |
| Molecular Visualization | PyMol | |

**Supplementary file 1e. Estimated secondary struture cntent of SiaD.**

| SiaD (μM) | 8 | | | | |
| --- | --- | --- | --- | --- | --- |
| SiaC (μM) | 0 | 4 | 8 | 12 | 16 |
| Helix (%) | 21.3 | 22.8 | 24.7 | 25.6 | 25.7 |
| Sheet (%) | 28.2 | 27.3 | 26.1 | 26.8 | 25.3 |
| Turn (%) | 12.8 | 12.7 | 12.4 | 12 | 12.5 |
| Other (%) | 37.7 | 37.2 | 36.2 | 35.6 | 36.4 |

Protein samples were tested in 10 mM Tris-HCl, pH7.5 and 500 mM NaCl in 0.1-cm-pathlength quartz cuvettes. The far-UV CD spectrum of protein samples were recorded in the range of 250-200 nm at 25 ^o^C, with 1-second/point scanning speed and 1-nm step. Three biological repeats were set for each sample. Three scans were averaged to obtain the final spectra for each sample. The raw data was processed using the software CDtoolX. The contribution of the buffer was substracted. As SiaC conformation has no significant change in SiaC-SiaD complex compared to its native structure, the CD signal of SiaC control was substracted from that of the corresponding SiaD-SiaC mixture sample to obtain the CD spectra of SiaD in mixture sample. A web-server BeStSel (Beta Structure Selection) was used for secondary structure determination.

**Reference**

Chen G, Gan J, Yang C, Zuo Y, Peng J, Li M, Huo W, Xie Y, Zhang Y, Wang T, Deng X, Liang H (2020) The SiaA/B/C/D signaling network regulates biofilm formation in *Pseudomonas aeruginosa*. *EMBO J* 39: e103412 10.15252/embj.2019103412 PMC7073463

West SE, Schweizer HP, Dall C, Sample AK, Runyen-Janecky LJ (1994) Construction of improved Escherichia-Pseudomonas shuttle vectors derived from pUC18/19 and sequence of the region required for their replication in *Pseudomonas aeruginosa*. *Gene* 148: 81-6

Yang C, Wu R, Liu H, Chen Y, Gao Y, Chen X, Li Y, Ma J, Li J, Gan J (2018) Structural insights into DNA degradation by human mitochondrial nuclease MGME1. *Nucleic Acids Res* 46: 11075-11088 10.1093/nar/gky855 PMC6237815
