# Supplementary material for: Structural basis for diguanylate cyclase activation by its binding partner in Pseudomonas aeruginosa
Source: eLife. 2021 Sep 9;10:e67289. doi: 10.7554/eLife.67289 (PMC8457831; doi:10.7554/eLife.67289)

Source data for Fig 1C

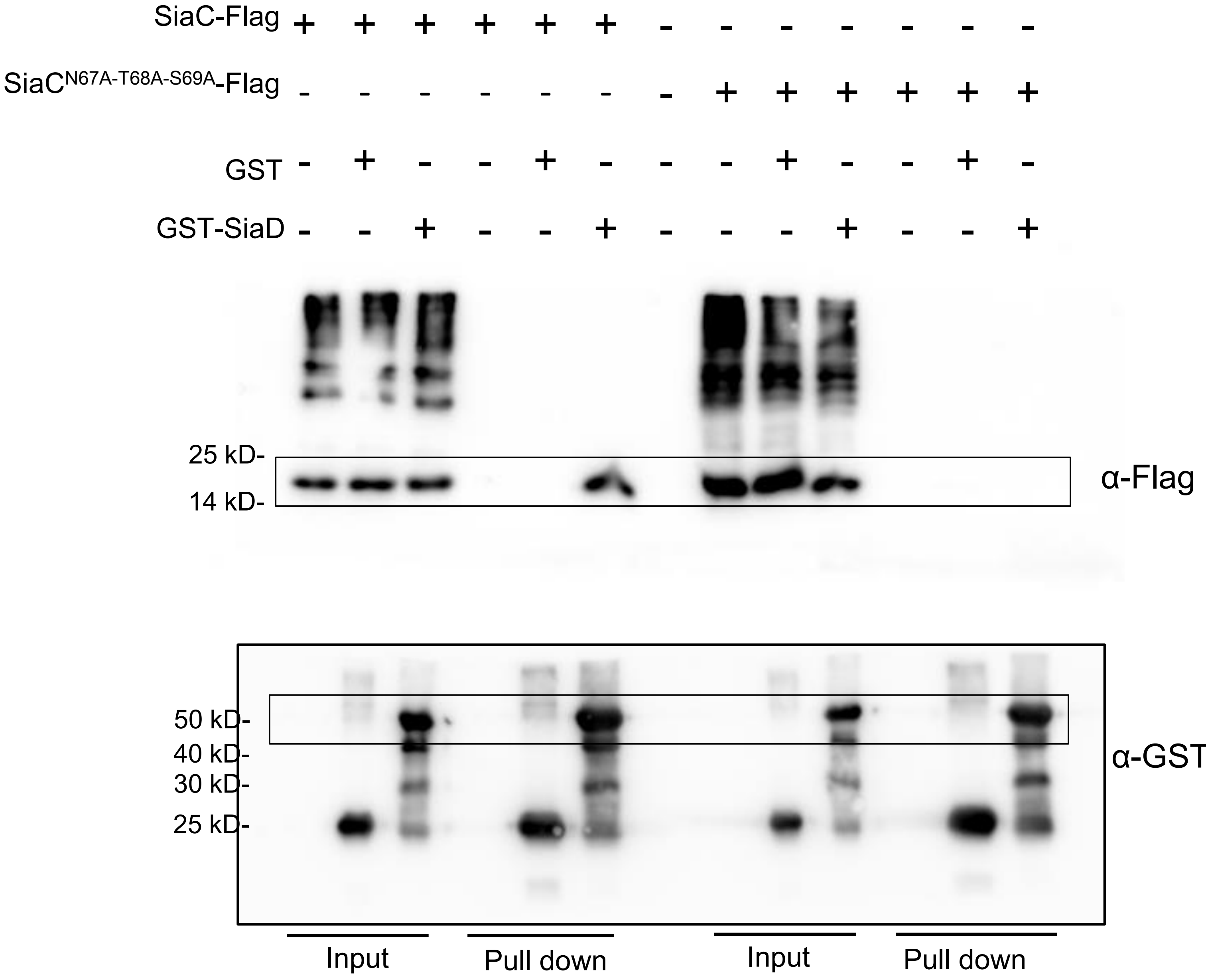

Source data for Figure supplement 1

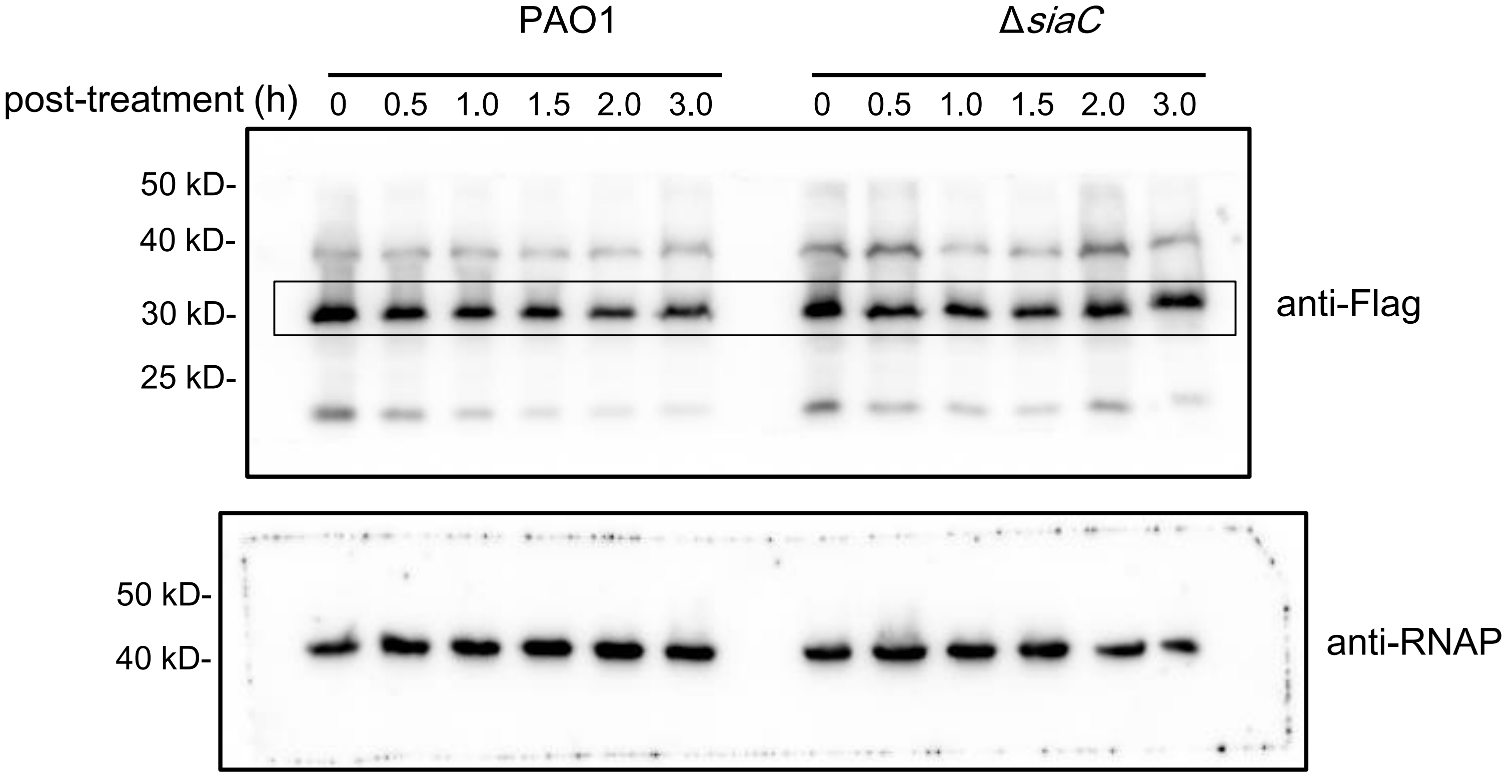

Source data for Figure supplement 2A

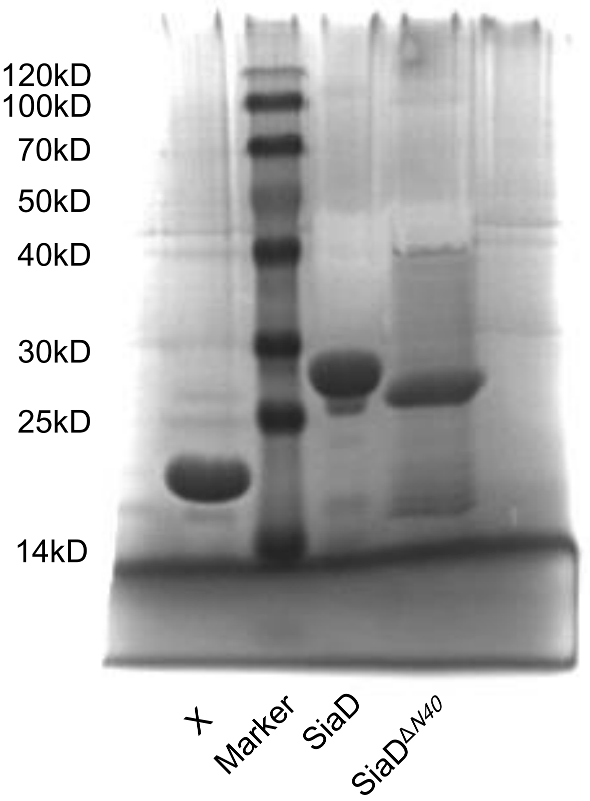

Source data for Figure supplement 8

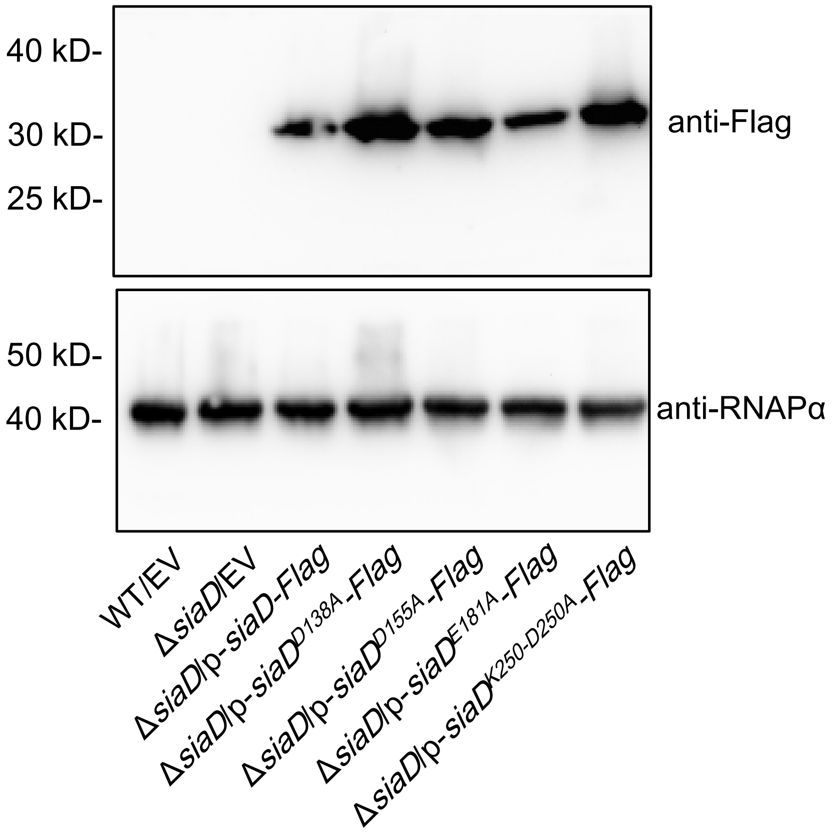

Source data for Figure supplement 15

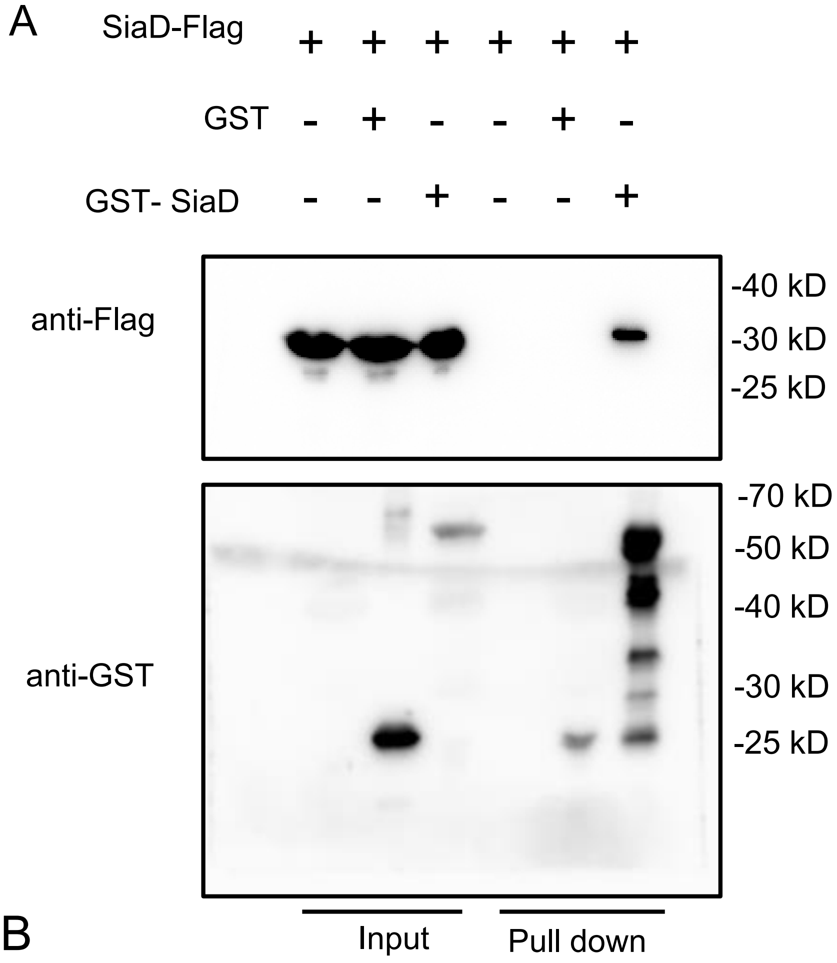

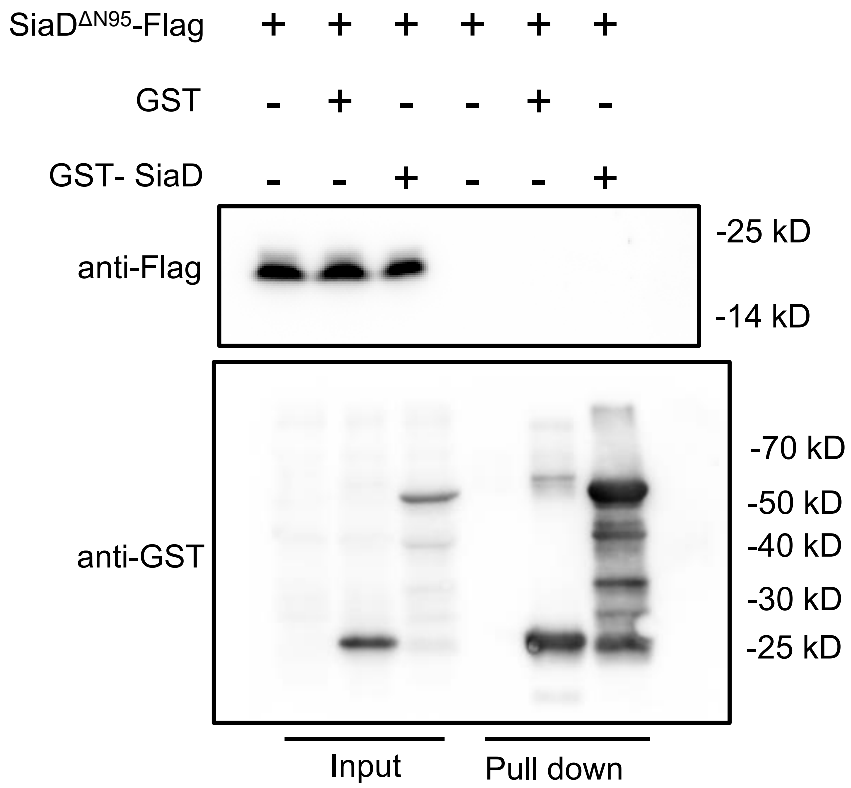

Source data for Figure supplement 16

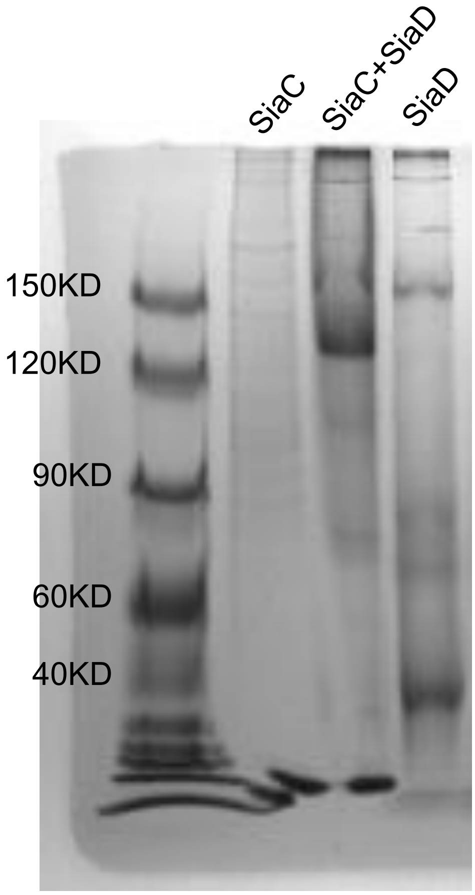

Supplement: Source data 1. [file elife-67289-supp2.pdf]
